# An innate ability: How do basal invertebrates manage their chronic exposure to microbes?

**DOI:** 10.1371/journal.ppat.1010897

**Published:** 2022-10-31

**Authors:** Leah M. Williams, Thomas D. Gilmore

**Affiliations:** Department of Biology, Boston University, Boston, Massachusetts, United States of America; University of Massachusetts, Worcester, UNITED STATES

## Abstract

Homologs of mammalian innate immune sensing and downstream pathway proteins have been discovered in a variety of basal invertebrates, including cnidarians and sponges, as well as some single-celled protists. Although the structures of these proteins vary among the basal organisms, many of the activities found in their mammalian counterparts are conserved. This is especially true for the Toll-like receptor (TLR) and cGAS-STING pathways that lead to downstream activation of transcription factor NF-κB. In this short perspective, we describe the evidence that TLR and cGAS-STING signaling to NF-κB is also involved in immunity in basal animals, as well as in the maintenance of microbial symbionts. Different from terrestrial animals, immunity in many marine invertebrates might have a constitutively active state (to protect against continual exposure to resident or waterborne microbes), as well as a hyperactive state that can be induced by pathogens at both transcriptional and posttranscriptional levels. Research on basal immunity may be important for (1) understanding different approaches that organisms take to sensing and protecting against microbes, as well as in maintaining microbial symbionts; (2) the identification of novel antimicrobial effector genes and processes; and (3) the molecular pathways that are being altered in basal marine invertebrates in the face of the effects of a changing environment.

## Introduction

**Fig 1:**
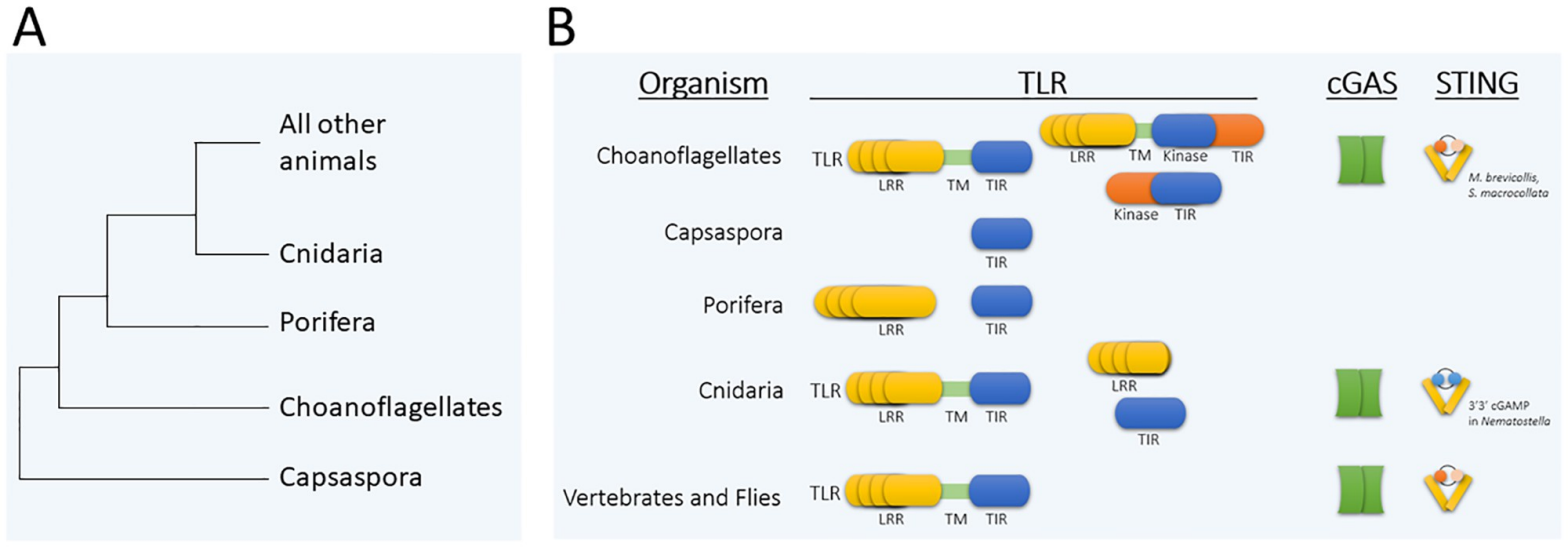
TLR and cGAS-STING signaling molecules in basal organisms. (**A**) General phylogenetic tree of the basal organisms discussed in this review. Capsaspora and choanoflagellates are protists. Porifera comprises the sponges, and Cnidaria includes sea anemones, jellyfish, corals, and hydra. (**B**) From the limited sampling of basal invertebrates, it is clear that there is great diversity in the TLR and cGAS-STING pathways, as compared to flies and vertebrates. For example, mammalian-like TLRs with extracellular pathogen recognition LRRs, TMs, and intracellular TIR domains are even present in some choanoflagellates. Nevertheless, there are also LRR- and TIR-only proteins in other phyla, including Cnidaria and Porifera. Similarly, cGAS and STING homologs are found among a variety of basal eukaryotes. However, these basal cGAS and STING homologs are sometimes missing domains found in vertebrates (e.g., for double-stranded DNA binding in cGAS or IRF3->interferon signaling in STING). The cyclic GAMP molecules preferred by STING can differ among basal organisms. See text for further details. LRR, leucine-rich region; TIR, Toll/interleukin-1 receptor domain; TLR, Toll-like receptor; TM, transmembrane domain.

Most of what we know about the response of organisms to microbial pathogens comes from the study of terrestrial animals, including notably insects and mammals. Terrestrial animals only sporadically encounter microbial pathogens. In contrast, marine organisms develop and live in a literal sea of microbes; indeed, the concentration of bacteria in shallow marine water is reported to be 1,000-fold higher than in air [1]. Therefore, one might expect that their antimicrobial immune systems would differ from terrestrial animals. In particular, continual exposure to microbes represents a special problem for basal organisms, such as sponges and sea anemones, which filter massive amounts of water through their bodies each day; however, few microbial pathogens have been identified for these filtering animals. The phylogenetic positions of the types of organisms that we discuss are shown in Fig 1A.

The molecular details of the 4 most prominent pathogen-sensing innate immune pathways in mammals—the Toll-like receptor (TLR), NLR, RIG-I-like receptor, and cGAS-STING pathways—are known in great detail. With advanced sequencing technologies, it has become clear that conserved components of these innate immune pathways are present in some of the simplest multicellular animals (e.g., anemones, corals, jellyfish, hydra, and sponges) and even some single-celled protists [2,3]. Although homologs of these innate immune pathway components are present in many basal organisms, they are generally reduced in number and complexity as compared to higher metazoans. However, whether these pathways are conserved in their molecular mechanisms and if and how they control immunity in these evolutionarily distant organisms are less clear.

Roles for TLR- or cGAS-STING-induced activation of NF-κB in basal immunity have been suggested by experiments in sea anemones, corals, hydra, sponges, and choanoflagellates [4–9]. Herein, we discuss some of the knowledge about the TLR and cGAS-STING pathways and roles that they may play in invertebrate immunity, as well as exciting information about approaches to antimicrobial immunity that may be gained from further study of basal immunity and its regulation.

### TLR signaling in basal innate immunity

In humans, there are 10 TLRs, which are transmembrane proteins at the cell surface or in intracellular organelles such as the endoplasmic reticulum, endosomes, or lysosomes. Mammalian TLRs, either directly or via associated molecules, recognize distinct pathogen components, often pathogen-associated molecular patterns (PAMPs), which include nucleic acids, lipids (e.g., lipopolysaccharide [LPS]), lipoproteins, and peptides) [10]. Engagement of the ligand by the TLR’s extramembrane leucine-rich region (LRR) initiates an intracellular TIR domain-mediated cascade (through the adapter proteins MYD88 and/or TRIF to a TRAF protein/MAPK pathway), which culminates in activation of important transcription factors (TFs) to initiate immune response gene expression. For mammalian TLRs, two such downstream TFs are AP-1 and NF-κB.

Two types of TLR-like homologs have been identified in the phyla Porifera (sponges) and Cnidaria (anemones, coral, hydra) [2,11,12] (Fig 1B). TLRs with prototypical bipartite structures consisting of an extracellular LRR joined via a transmembrane domain to an intracellular TIR domain have been found in many basal metazoans, although such organisms generally have only single copies of such genes [2,5]. The cnidarian LRRs have multiple cysteine clusters similar to what is found in several other invertebrates [2]. On the other hand, prototypical bipartite TLRs have not been found in sponges [13,14] or several cnidarians, such as *Hydra vulgaris* and the sea anemone *Aiptasia pallida*, where the external LRR and internal TIR-like domains are expressed as separate proteins encoded by distinct genes.

Several mammalian TLR intracellular downstream signaling components, including MYD88, TRAF, TAK, and IκB, have homologs in both Porifera and Cnidaria, and these downstream proteins appear complete in structure as compared to their mammalian counterparts [15]. Similarly, homologs of the mammalian innate immune-activated TFs do exist in basal invertebrates, although they are again generally reduced in number in these organisms. For example, whereas humans have 5 NF-κB-like proteins, only single NF-κB proteins are found in cnidarians, poriferans, and some protists [6,7,16–20]. AP-1-like proteins have been identified in poriferans and cnidarians, but there is little data regarding their biological functions and regulation in these organisms. In the single-celled protist *Capsaspora owczarzaki*, no obvious TLR proteins have been found; however, *Capsaspora* does contain homologs to some TLR pathway intracellular signaling components and to NF-κB [18,21]. On the other hand, many single-celled choanoflagellate species have multiple TLR-like proteins with the following types of structures: (1) prototypical, mammalian-like joined LRR-TIR domain proteins; (2) a transmembrane protein with an extracellular LRR and an internal kinase domain; and (3) some TIR-only domain proteins [22]. However, the LRRs of these choanoflagellate TLR-like proteins do not have clear cysteine-rich clusters that are found in TLRs of all other phyla. No AP-1 homologs have been identified in organisms basal to sponges, whereas NF-κB homologs are present in *Capsaspora* and many choanoflagellate species [3].

The pathogen recognition abilities of basal TLRs appear to differ from mammalian TLRs. Notably, Gauthier and colleagues [23] demonstrated that about 40 gram-negative marine microbes, as well as their purified LPS molecules, are not detected by mammalian LPS receptor systems, suggesting a lack of coevolution between terrestrial and marine microbes. Furthermore, these marine PAMPs contain longer acyl side chains on the lipid portion of their LPS [23]. It is tempting to speculate that this difference in marine microbes has led to the evolution of single TLRs in basal marine invertebrates that have promiscuous abilities to recognize a variety of microbes, or perhaps, that there are other unknown types of sensing molecules in basal marine organisms that recognize this diversity of bacterial PAMPs. On the other hand, at least one species of hydra has hundreds of extracellular TLR-like LRRs, but a limited number of internal TIR-only signaling domains [24], raising the possibility that their numerous extracellular domains can bind to many PAMPs, but then signal downstream through a limited number of intracellular effector domains. The ability of basal TLRs to signal to NF-κB appears to be highly conserved across phyla. For example, expression of separate hydra LRR and TIR-only proteins or a sea anemone bipartite TLR in human cells can lead to activation of the endogenous human NF-κB pathway by treating those cells with either a coral bacterium or flagellin [5,11]. Moreover, the internal TIR domains of a sea anemone and coral TLR can directly interact with the human adapter MYD88 [5,7]. It has yet to be determined whether basal TIR domain proteins contain enzymatic activities, as have been described for TIR proteins from several other types of organisms [25].

Treatment of coral [26] and sponge [7] tissues with conventional *E*. *coli* LPS results in both increased NF-κB DNA-binding activity and up-regulation of mRNA-encoding NF-κB pathway members. Moreover, TLR signaling is proposed to have a role in bacterial defense in the sea anemone *Nematostella* and *H*. *vulgaris* [4,5]. For example, in *Nematostella*, a phylum-specific circulating organelle, the nematosome, is capable of engulfing pathogenic bacteria, and cells in the nematosome express TLR->NF-κB signaling homologs (as well as cGAS-STING; see below) [5]. In *H*. *vulgaris* polyps, knockdown of MYD88 results in patterns of differential host gene expression as well as pathogen susceptibility that suggest a role for TLR signaling in sensing both commensal and pathogenic microbes [4]. Taken together, the data suggest that TLRs from basal organisms can respond to external PAMP signals, but may have less specialized, or even undiscovered ways, of detecting a large variety of microbial molecules with properties that are distinct from mammalian microbes.

### cGAS-STING signaling in basal immunity

In mammals, the cytosolic cGAS-STING pathway recognizes and is activated by cytosolic double-stranded DNA. That is, cGAS binds to free DNA, which triggers the formation of 2′3′ cGAMP. This second messenger then binds to STING, which activates the kinase TBK1, leading prominently to downstream activation of TF IRF3 for antiviral expression of β-interferon and other coregulated genes. Homologs of cGAS and STING have been identified in cnidarians [27] and some choanoflagellates [22,28]. However, whether basal cGAS proteins can directly bind to DNA is unclear [27].

The sea anemone *Nematostella* has a STING homolog with a three-dimensional structure that is quite similar to human STING [27]. Although the anemone STING can bind 2′3′ cGAMP indistinguishably from human STING, the *Nematostella* cGAS appears to produce a 3′3′ cGAMP that anemone STING recognizes through nucleobase-specific contacts not observed in human STING [27]. However, the *Nematostella* STING lacks the residues needed for downstream activation of IRF3, and indeed, interferons are not present in basal metazoans, indicating that any cnidarian immunity driven by cGAS-STING acts through different downstream immune effector molecules. Of note, Margolis and colleagues [8] have shown that bacterial infection or treatment with a 2′3′ cGAMP mimetic can activate an antipathogen gene expression response in *Nematostella*, which also leads to up-regulation of nuclear NF-κB expression. Moreover, STING has been shown to play a role in an antibacterial response in some choanoflagellates [9].

### NF-κB as a downstream effector of basal immunity

Overall, roles for TLR- or cGAS-STING-induced activation of NF-κB for basal immunity have been suggested by experiments in *Nematostella*, corals, hydra, sponges, and choanoflagellates [4–8], Nevertheless, some basal organisms, such as some choanoflagellate species and ctenophores, do not appear to have NF-κB homologs. Most basal animals have single NF-κB proteins that have either the general bipartite structure of mammalian NF-κB p100 proteins—with an N-terminal DNA-binding/dimerization domain and a C-terminal ankyrin repeat inhibitory domain—or these two domains of NF-κB are encoded by separate genes [3].

**Fig 2:**
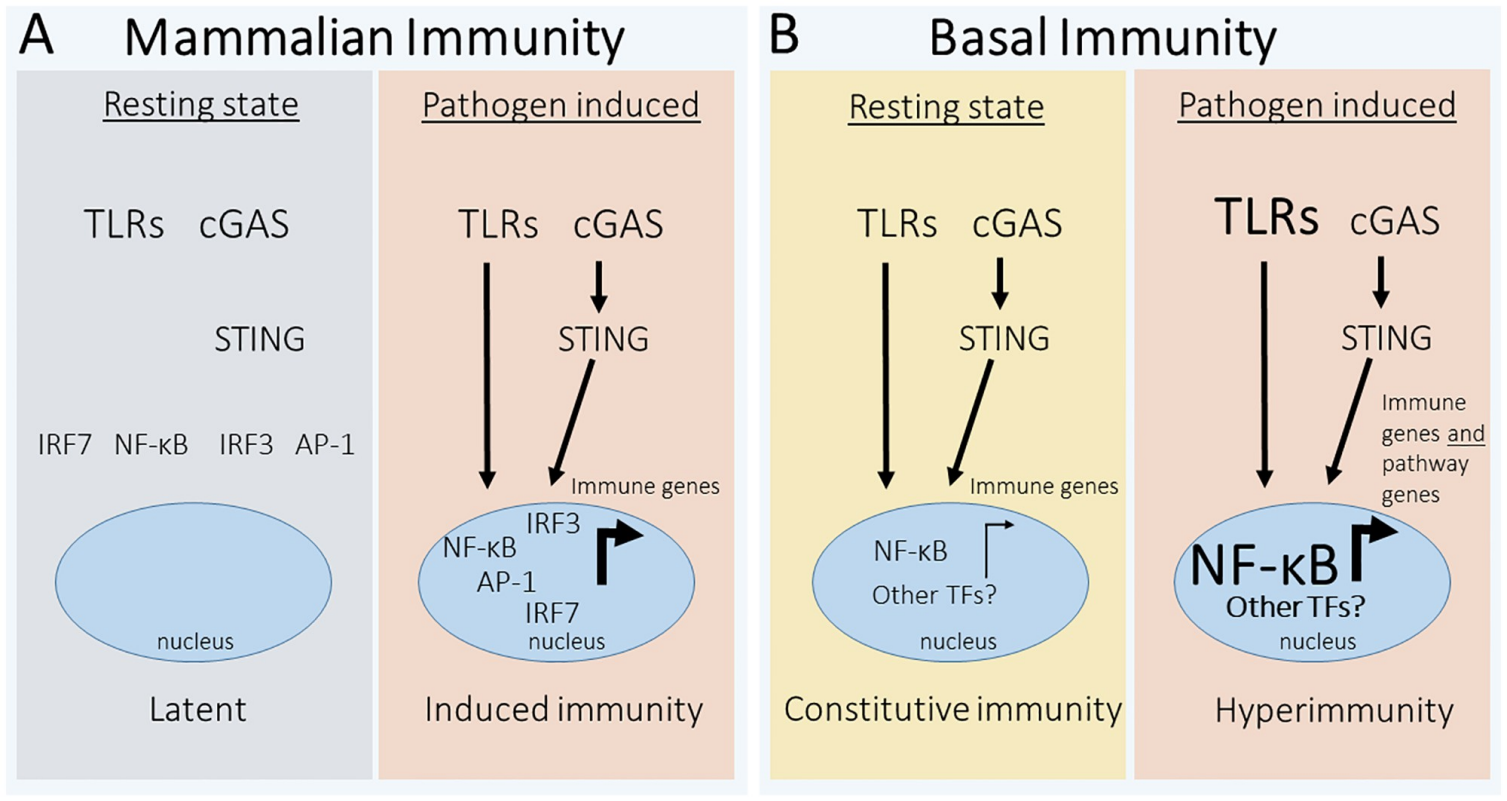
Mammalian and basal innate immune signaling via NF-κB. (**A**) In a simplified mammalian immune pathway, the innate immune pathway is OFF in the resting state, especially as indicated by no nuclear NF-κB-dependent transcription of immune genes. In the pathogen-induced state—either via activation of TLRs or cGAS-STING—the nuclear translocation of active NF-κB leads to rapid and robust activation of immune effector genes. (**B**) In many basal invertebrates, in the resting state, it appears that there is constitutive activation of the NF-κB pathway, either due to resident microbes or presumably chronic exposure to microbes in marine environments. An acute exposure to pathogens can lead to a hyperimmune state, which is characterized by further nuclear translocation of NF-κB, as well as increased transcription of innate immune pathway components (indicated by the larger TLR and NF-κB fonts in the right half of the figure). Further details can be found in the text.

Upon activation of mammalian noncanonical NF-κB signaling, the C-terminal domain of p100 is phosphorylated by an IκB kinase and this phosphorylation promotes proteasome-dependent processing of p100 to the active N-terminal p52 protein, which then enters the nucleus to affect gene expression. Bipartite NF-κB proteins of corals, anemones, sponges, and the protist *Capsaspora* can undergo proteasome-dependent processing and nuclear translocation when expressed in mammalian cells in culture [6,7,16,18]. However, it is still not clear whether and under what circumstances such induced processing of NF-κB occurs in the native animals. That is, in the sea anemone *Aiptasia* and one sponge, most NF-κB is in its processed form under apparently resting conditions [7,16]. Furthermore, treatment with LPS or loss of a symbiont induces transcriptional activation of NF-κB and its pathway components in one coral and in the anemone *Aiptasia* [6,16]. Thus, in contrast to what is seen with mammalian-induced innate immunity, NF-κB in many basal organisms appears to be in a constitutively active, nuclear state under most circumstances, and further induction of the pathway may proceed by transcriptional induction (rather than posttranslational processing) of NF-κB and its upstream pathway components (Fig 2).

### Other biology of the basal innate immune pathways

In addition to their roles in immunity, TLR->NF-κB pathways appear to play species-specific roles in early development in many basal organisms [2,3]. Roles for TLRs and NF-κB in development have been suggested by experiments in the anemone *Nematostella*, the sponge *Amphimedon queenslandica*, and the protist *Capsaspora* [5,18,29]. In *Nematostella*, knockdown of its single TLR results in embryos that fail to gastrulate [5], and knockdown of NF-κB results in lack of development of the immune stinging cells (cnidocytes) present in members of this phylum [30]. In the sponge *Amphimedon*, NF-κB transcripts are expressed throughout the early embryo shortly after cleavage and then primarily in large granular cells in later stage embryos [29]. In *Capsaspora*, NF-κB expression and DNA-binding activity levels vary in different life stages, suggesting specific roles for NF-κB in each life stage [18]. We wish to point out, however, that the early developmental roles of TLRs and NF-κB in many organisms appear to be independently derived activities, where the pathways and molecules have been retooled for specific purposes in many organisms and these developmental activities have not been retained through evolutionary history.

### Conclusions and future considerations

As evident from this discussion, there is still much to be learned about the molecular details and effector molecules of basal invertebrate immunity. Unlike terrestrial animals, which only sporadically encounter pathogens from air or through a layer of skin, filtering invertebrates, which continuously pass massive amounts of seawater through their bodies, might require some constant level of immune protection. Thus, one notable paradigm shift is that many marine invertebrates may have constitutively active levels of NF-κB-induced immunity, likely to deal with the soup of pathogens these filtering animals encounter on a routine basis. Indeed, the overall gene expression pattern of *H*. *vulgaris* polyps with TLR signaling disrupted by antisense reduction in MYD88 have an overall gene expression pattern that overlaps considerably with polyps grown in bacteria-free conditions, suggesting that exposure to normal bacterial flora activates the TLR pathway [4]. In addition, the sponge *Halichondria panicea*, which maintains a low abundance of bacteria, has constitutively high expression of TLR->NF-κB pathway genes [31]. Furthermore, NF-κB is constitutively nuclear in the anemone *Aiptasia*, even though the levels of nuclear NF-κB can be modulated by algal symbionts [16]. If, indeed, marine invertebrates do have high levels of basal immunity, they must also have mechanisms to avoid the harmful effects of chronic immunity that are seen in mammals. Nevertheless, basal organisms do appear to have a second level of induced “hyperimmunity,” which can be controlled by transcriptional induction of immunity genes and pathways. Regardless of the details, our mammalian-centric view of latent and induced innate immunity may not completely apply to basal marine invertebrates.

Further research into the potentially unique immunity of basal organisms might (1) uncover novel antimicrobial effector molecules (encoded by target genes of NF-κB or other immunity TFs), as many basal genomes contain hundreds of genes with no known homologs; and (2) provide an understanding of how our changing oceans (namely from climate change) are affecting immunity for invertebrate health, including notably corals and their emerging pathogen diseases.
